# Molecular Thermodynamics Using Nuclear Magnetic Resonance (NMR) Spectroscopy

**DOI:** 10.3390/inventions4010013

**Published:** 2019-02-21

**Authors:** Viswanathan V. Krishnan

**Affiliations:** 1 Department of Chemistry, California State University, Fresno, CA 93740, USA; 2 Department of Pathology and Laboratory Medicine, School of Medicine, University of California, Davis, CA 95616, USA

**Keywords:** NMR, thermodynamics, chemical exchange, Eyring equation

## Abstract

Nuclear magnetic resonance (NMR) spectroscopy is perhaps the most widely used technology from the undergraduate teaching labs in organic chemistry to advanced research for the determination of three-dimensional structure as well as dynamics of biomolecular systems... The NMR spectrum of a molecule under a given experimental condition is unique, providing both quantitative and structural information. In particular, the quantitative nature of NMR spectroscopy offers the ability to follow a reaction pathway of the given molecule in a dynamic process under well-defined experimental conditions. To highlight the use of NMR when determining the molecular thermodynamic parameters, a review of three distinct applications developed from our laboratory is presented. These applications include the thermodynamic parameters of (a) molecular oxidation from time-dependent kinetics, (b) intramolecular rotation, and (c) intermolecular exchange. An experimental overview and the method of data analysis are provided so that these applications can be adopted in a range of molecular systems.

## 1 Introduction

Starting from the time when Rabi et al. demonstrated the first nuclear magnetic resonance (NMR) spectrum in 1938, the dramatic evolution of the technology has been unmatched over the years and thus deserving of encyclopedias to cover the methods and applications [1,2]. The measurement of dynamic processes by NMR has been an integral part of the progress, particularly with the development of Fourier transform NMR (FTNMR) [3] and subsequently the two-dimensional NMR techniques such as the exchange-correlation spectroscopy (EXSY) [4]. The concepts and applications of dynamic NMR spectroscopy that are fundamental in determining the thermodynamic parameters have been covered in excellent monographs, as well as review articles [5–8]. This article provides a review of applications along with the underlying theory and experimental aspects in determining the molecular thermodynamic parameters.

## 2 Nuclear Magnetic Resonance (NMR) Spectroscopy

NMR spectroscopy is widely used in many areas of science from classrooms to advanced research laboratories. However, a brief introduction to the fundamental concept could be considered appropriate for the audience of this journal. Quantum mechanics defines *spin* as an intrinsic property of sub-atomic particles. In a classical mechanics-based description, the precision of the net nuclear magnetic moment around an externally applied magnetic field is defined as the Larmor frequency of the nucleus. This approach further extends the description of the NMR spectroscopy as follows: when a sample containing magnetically active nuclei is placed into a strong magnetic field, and a radio frequency (RF) radiation is applied, a resonance absorption of incident radiation occurs at the frequency corresponding to the nuclear spin precession. Although valid for a single nucleus, a classical description is inadequate for describing a multitude of spin-spin interactions.

A quantum mechanical description is essential in order to understand the concepts of NMR spectroscopy. In an external magnetic field, the degeneracy between the nuclear spin states is removed with the energy level separation between the states being directly proportional to the applied magnetic field. The energy level difference leads to a resonance absorption in the radio frequency range. The resonance frequency of the nuclei (measured in ppm) is highly sensitive to the local magnetic properties of the nuclei, and thus an NMR spectrum is a footprint of the molecular configuration. Typically, an NMR spectrometer is defined by the resonance frequency of hydrogen atoms (protons). For example, a 400-MHz NMR spectrometer means that the magnetic field at which the protons would produce resonance is at this frequency (ν = γhBo/2π; γ is the gyromagnetic ratio of the proton, *h* is Planck’s constant, and *Bo* is the applied magnetic field). For example, all of the experimental data presented in this article were all collected in a 400-MHz NMR spectrometer. Although NMR spectra can be obtained in solid or liquid phases, the solution-state NMR spectroscopy generally focuses on the spin $\frac{1}{2}$ nuclei such as ^1^H, ^15^N, ^13^C, and ^31^P, because of the simplicity in the spectrum. The basis of NMR spectroscopy is to apply radio frequency pulses, which perturb the population difference between the levels of the spin states $\left(\pm \frac{1}{2}\right)$ and follow the relaxation response in the form of a free induction decay (FID).

Fourier transformation collected at this time-domain signal provides a linear spectral response (i.e., the NMR spectrum) of the sample. A typical one-dimensional NMR spectrum identifies the chemical nature of the spin in the molecular structure in the form of a ‘chemical shift’, as well as the number of such spin systems present in the molecule. The second most crucial NMR parameter is due to the indirect coupling between the nuclear spins through the electrons (hyperfine interactions), which introduces splitting in the observed chemical shifts, depending on the number of coupled spins in a particular network of spins. During the non-radiative relaxation process, a multitude of interactions aid the spin states back to the equilibrium population. The systematic experimentation of these events is the basis of measuring the kinetic and thermodynamic processes of a molecular system.

When a one-dimensional NMR spectrum is obtained and contains too many overlapping peaks to permit the identification of individual resonances, multi-dimensional NMR techniques that enable the resonances to be spread out in two or more dimensions are routinely used. For example, in a two-dimensional NMR spectrum, there are two frequency axes, which can correspond to the same kind of nuclei (i.e., ^1^H–^1^H, homonuclear), with the third dimension representing peak intensity. These multi-dimensional and higher-dimensional spectra can be interpreted to yield sequential or long-range connectivity between hydrogen atoms that are either covalently connected (correlation spectra) or are near each other (nuclear Overhauser effect/enhancement spectroscopy, NOESY) in the molecule. There is a wide range of excellent textbooks for the specific needs of the user, as well as scholarly research journals that can be found to enhance particular goals.

## 3 Chemical Exchange and Kinetics

The rate of a chemical reaction is mostly a function of the thermodynamic state of a system: the concentration of the various species, pressure, and more importantly, the temperature. For example, in the case of an elementary reaction (A↔B, in which both arrows represent an equilibrium process), the dependence on the rate of concentration is defined regarding a rate constant *k* (*Rate* = *k*[*A*]). The equilibrium process between the reactants (A) and product (B), which was measured in the NMR spectrum, is typically referred to as a *chemical exchange*. During the chemical exchange process, an individual nucleus from the reactant moves to a different environment in the product, and thus manifests a change in the NMR spectrum. With the change in state of the system, a direct change in the spectrum is observed; thus, extracting relevant NMR parameters enables an estimation of the thermodynamic parameters of the reaction process.

A phenomenological description of the chemical exchange process between two states of molecule A and B can be written as:

(1) $$A\overset{k_{1}}{\underset{k_{-1}}{⇄}}B$$

where the change in the concentrations of A and B can be related via the rate equations:

(2) $$\frac{d\left[A\right]}{dt}=-k_{1}\left[A\right]+k_{-1}\left[B\right]\;\frac{d\left[B\right]}{dt}=k_{1}\left[A\right]-k_{-1}\left[B\right]$$

Equation (2) is rewritten in a matrix form as:

(3) $$\frac{d}{dt}\left[\begin{array}{c} \left[A\right]\\ \left[B\right] \end{array}\right]=\left[\begin{array}{cc} -k_{1} & k_{-1}\\ k_{1} & -k_{-1} \end{array}\right]\left[\begin{array}{c} \left[A\right]\\ \left[B\right] \end{array}\right]$$

If the rate of decay of [A], or an increase of [B], is slower than the rate at which the NMR signal is acquired, it would then be possible to follow the reaction process in real time. The coupled equations (Equation (3)) can be solved to determine how the molecular system completes the reaction process. By defining the deviation of the population of the given molecular species from equilibrium at any given time $(\Delta \left[A\right]\left(t\right)=\left(\left[A\right]-{\left[A\right]}_{eq}\right)$, the time dependence can be written as:

(4) $$\Delta \left[A\right]\left(t\right)=\Delta \left[A\right]\left(0\right)e^{-k_{ex}t}$$

where *k*_*ex*_ = *k*_1_ + *k*_*−*1_. One of the examples presented in this manuscript (ibid IV.1) demonstrates the real-time monitoring of the reaction rate in the case of an oxidation process, under equilibrium conditions (the rate of the forward and reverse reactions are equal), leading to:

(5) $$\frac{d\left[A\right]}{dt}=\frac{d\left[B\right]}{dt}=0\Rightarrow k_{1}{\left[A\right]}_{eq}-k_{-1}{\left[B\right]}_{eq}=0\;\text{or}\;{\text{K}}_{eq}=\frac{k_{1}}{k_{-1}}=\frac{{\left[B\right]}_{eq}}{{\left[A\right]}_{eq}}$$

In the NMR spectrum acquired under equilibrium conditions, the spectral lines are directly proportional to the population of the particular molecular species in the sample. In the above example, the molecular system can either be the same molecule undergoing a conformation change (two different states that are distinguishable in the NMR spectrum) leading to an *intramolecular* exchange or two different molecules suggesting an *intermolecular* exchange. In this article, examples of both intramolecular exchange (ibid IV.2) and intermolecular (ibid IV.3) exchange are presented.

## 4. Molecular Thermodynamics

The quantitative NMR spectra, besides providing real-time analysis of the reaction process, offers significant insight into the molecular structure. The reaction process that is depicted as a chemical exchange in thermodynamic terms relates the state of the molecular system passing through a transition state, which is defined by a kinetic barrier (activation energy). The kinetic barrier can be determined by measuring the reaction rates as a function of the system parameters, such as temperature. The temperature dependence of the reaction rate, which is empirically described by the Arrhenius equation ([9]), is as follows:

(6) $$k_{ex}=A\exp\left(-\Delta E^{‡}/RT\right)$$

In Equation (6), *A* is defined as the pre-exponential factor, *R* is the gas constant (8.314 J/ (K*·*mol)), *T* is the sample temperature in Kelvin, and ∆*E*^‡^ is the activation energy to the transition state. The Arrhenius formalism was later refined by Eyring, which allows the definition of ∆*E*^‡^ to be in terms of Gibbs energy (∆*G*^‡^ ), enthalpy (∆*H*^‡^ ), and entropic (∆*S*^‡^ ) contributions of the transition state as follows [10]:

(7) $$k_{ex}=\frac{k_{B}T}{h}\exp\left(\Delta G^{‡}/RT\right)\;\;\;=\frac{k_{B}T}{h}\exp\left(-\frac{\Delta H^{‡}}{RT}+\frac{\Delta S^{‡}}{R}\right)$$

where *k*_*B*_ is the Boltzmann constant (1.3807 × 10^−23^ J/K), *h* is Planck’s constant (6.62697 × 10^−34^ J*·*s), and *R* is the gas constant.

Furthermore, when describing the temperature dependence of the rate, the equilibrium process (Equation (5)) is dependent on the system parameters. The temperature dependence of the difference in the Gibbs energy between the reaction and product is given by:

(8) $$\Delta G=\Delta G^{‡}+RT\ln\left(K_{eq}\right)$$

where ∆*G*^‡^ is the Gibbs energy at standard conditions. Under equilibrium conditions (∆*G*^‡^ = 0), Equation (8) becomes:

(9) $$\Delta G^{‡}=-RT\ln\left(K_{eq}\right)\;K_{eq}=\exp\left(-\Delta G^{‡}/RT\right)$$

Equation (9) has a close resemblance to the Arrhenius equation (Equation (6)), except for the pre-exponential factor.

In order to determine the thermodynamic parameters, the experimental results can be analyzed by collecting the data as a function of temperature. The plot of inverse temperature (1/*T*) versus the natural logarithm of (*k*_*ex*_/*T*), which is known as the Eyring plot, is often utilized to determine the thermodynamic parameters [10]. An example of the Eyring plot is presented later in the application (for an example, see later in Figure 2c). The slope and the intercept of the Eyring plot relate to the activation enthalpy and entropy:

(10) $$slope=-\Delta H^{‡}/R\;\text{intercept}=\ln\left(k_{B}/h\right)+\Delta S^{‡}/R$$

The constant term in the intercept is given as 23.76024 (1/*Ks*) (natural logarithm of *k*_*B*_/*h*). The Eyring plot is an approach that is broadly used to determine the thermodynamic parameters using NMR spectroscopy. As pointed out by Lente et al. [11], there is a widespread view among some of the researchers that “*the value of the entropy of activation is unreliable, because it is calculated by extrapolation to infinite temperature*.” This statement is based on the linearized form of Equation (10):

(11) $$\ln\left(\frac{k_{ex}}{T}\right)=-\frac{\Delta H^{‡}}{RT}+\frac{\Delta S^{‡}}{R}+\ln\left(\frac{k_{B}}{h}\right)$$

When *ln*(*k*/*T*) is plotted versus (1/*T*), the enthalpy and entropy of activation are determined from the slope and intercept, respectively (Equation (10)). Anecdotally, it is suggested in some literature that as in Eyring’s description, the determination of the entropy relates to the intercept at (1/*T*) = 0 *or T* = ∞, and thus the extrapolation to infinity consequently leads to the unreliable estimation of the activation entropy. In the modification proposed by Lente et al. [11], Equation (11) is rewritten in a different form (multiplying both sides of Equation (11) by T) as:

(12) $$\ln\left(\frac{k_{ex}}{T}\right)=-\frac{\Delta H^{‡}}{RT}+\frac{\Delta S^{‡}}{R}+\ln\left(\frac{k_{B}}{h}\right)$$

In this form of the equation, a plot of *T* versus $T\times ln\left(k_{ex}/T\right)$ will provide an estimate of the entropy from the slope and the enthalpy from the intercept. A statistical analysis from the two different approaches (equations (11) and (12)) further suggests that the error estimates from these methods have similar numerical precision. Either of the approaches will yield reliable estimates of the enthalpy and entropy values, as long as the experiments are designed to collect data over a range of temperature values with meaningful sampling.

## 5. Applications

The use of NMR spectroscopy to determine molecular thermodynamic parameters has been widely used across many disciplines of biology, chemistry, chemical engineering, physics, and structural biology. Indeed, outstanding reviews are covering both the methodology and applications by many experts [12–15]. However, in this section, the motivation is to summarize the results obtained in a moderately equipped research university, and more importantly, undergraduate students performed all of the experiments. Three examples are presented. In the first example, real-time NMR experiments are used to determine the rate of oxidation in L-cysteine ester, and the temperature-dependent rates are then used to estimate the Gibbs energy of oxidation. The second and third examples are used to demonstrate the use of molecular thermodynamic parameters in an intramolecular chemical exchange and intermolecular chemical exchange mechanisms, respectively. The intramolecular exchange phenomenon is related to the partial amide bond rotation modified in *m*-DEET (*N,N*-diethyl-*m*-toluamide). The intermolecular exchange mechanism follows the quasi-equilibrium process between the neurotoxin BMAA (β-*N*-methylamino-L-alanine) and bicarbonate ions that led to the simultaneous presence and chemical exchange between BMAA and its carbamate adducts in solution.

### 5.1 Thermodynamics of Parameters of the Oxidation Process in Model Cysteine Molecules

Oxidation is a fundamental reaction in chemistry and biology. In particular, the thiol group in cysteine (Cys) is integral to the protein structure and many biological functions. Polar solvents, such as a dimethyl sulfoxide (DMSO), are known to oxidize the thiol groups in L-Cys molecules to form a disulfide bond [16–23].

**Figure 1:**
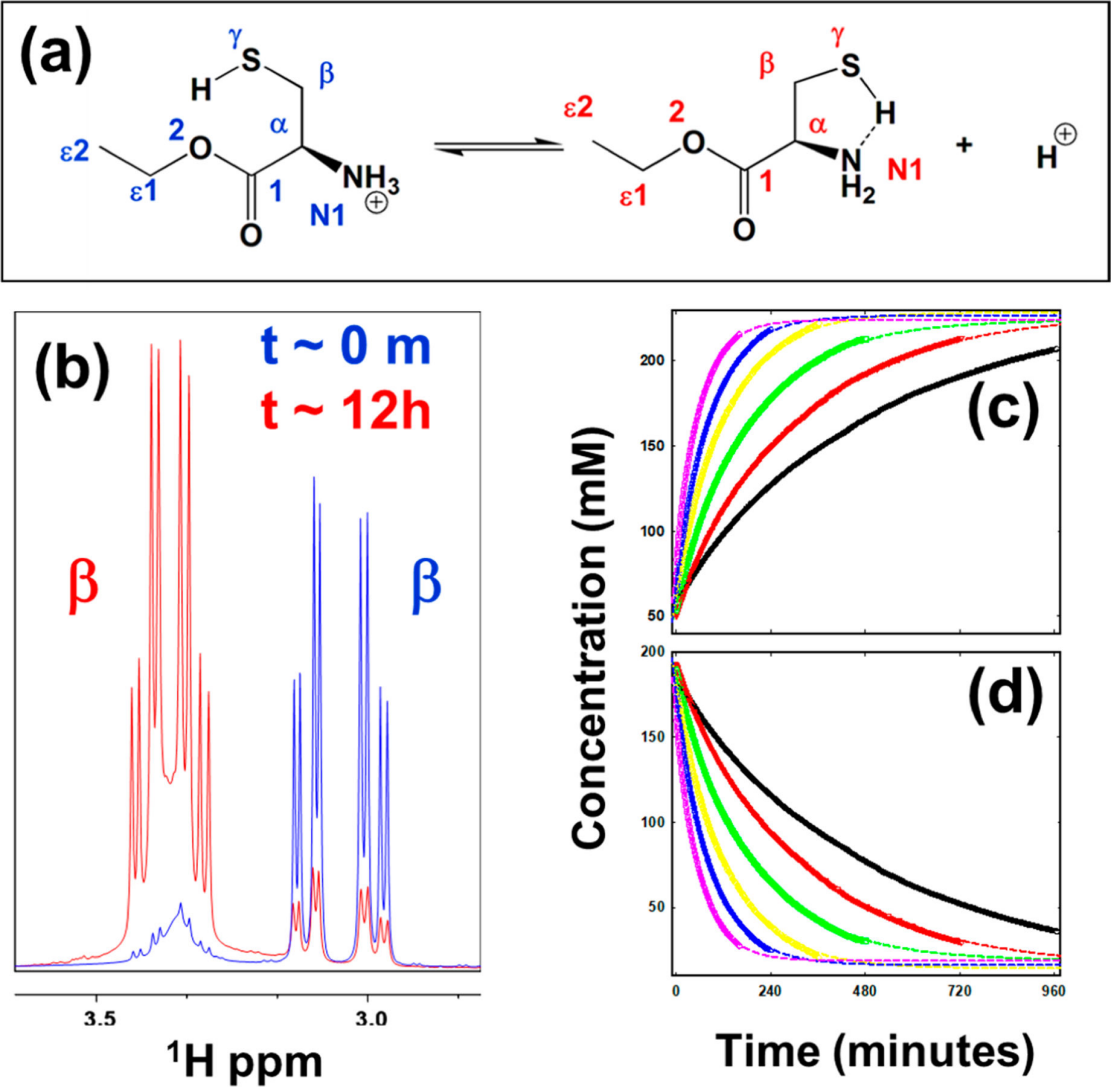
Determination of molecular thermodynamic parameters from the rate of oxidation. **(a)** Structural change due to the oxidation of the thiol proton in the model system L-cysteine ethyl ester ([CysEE-H] +). **(b)** Chemical shift changes due to the oxidation-mediated changes in L-cysteine ethyl ester in the sub-spectral region corresponding to the β -protons of the reactant (blue) and that of the product (red) obtained in dissolving the DMSO-d_6_. The first-order kinetics of the reaction process of L-CysEE at various temperatures are also shown. **(c)** The reduction of the reactant ([CysEE-H] +) measured directly by NMR for various sample temperatures, and the corresponding increase of the ionized product ([CysEE]) **(d).** The experimental measurements are shown as symbols, and the non-linear least square fit to the respective experimental data are shown as dashed lines (samples temperatures are given in the legends). Figure reproduced with permission [24].

The experimental details regarding the measurement of the kinetic parameters of the oxidation process have been described previously [24]. Figure 1 (panel a) shows the oxidation reaction in L-Cys ethyl ester (EE). Furthermore, the observed oxidation-mediated change was slow enough to be monitored in real-time using NMR spectroscopy. This resulting change is much slower than the characteristic time of the typical NMR experiment and leads to significant changes in the NMR spectrum that could be measured as a function of time. The NMR spectra in Figure 1b highlights the effect of the oxidation-mediated changes in the case of L-CysEE on the chemical shifts of the ^1^Hβ protons. The spectra plotted in blue are at the beginning of the experiment, while the ones plotted in red are after the reaction at 30 *°*C. The ^1^Hβ protons of the oxidized form shift downfield by 0.3 ppm, and therefore, the area under the curve of each of them is used to quantify the reactants and products.

In this oxidation reaction, the experimental conditions favor the formation of the product, and therefore the reverse reaction can be ignored $\left(k_{1}\gg k_{-1}\right)$ in Equation (1)). Also, to accommodate the presence of the solvent, the oxidation-mediated change is modeled as a *pseudo*-first-order reaction process. In this cose, the rate equation (Equation (2)) becomes:

(13) $$\frac{d\left[C\right]}{dt}=k\prime \left[C\right]\left[DMSO\right]$$

where [C] can be considered the oxidized species. Upon rearranging Equation (13) and defining a *pseudo*-first-order rate constant $k_{ex}=k’\times \left[DMSO\right]$, the simplified rate equation becomes:

(14) $$\frac{d\left[C\right]}{dt}=k_{ex}\left[C\right]$$

Equation (14) can be used to describe the kinetics by virtue of the total concentration of *C* remaining the same, and there are no other oxidation processes due to the presence of residual water since DMSO is a hygroscopic solvent) during the measurement time of the kinetics. The concentration of reactant and product were calculated according to:

(15) $$\left[C\right]=\frac{\left[A\right]}{\left[B\right]}{\left[C\right]}_{0}$$

where [A] is the area of the NMR signal of the reactant, [B] is the total area of the NMR signals from the product, and [C]_0_ is the initial concentration of the reactant at each temperature. The solution to Equation (15) is given by (see also Equation (4)):

(16) $$\left[C\right]={\left[C\right]}_{0}e^{-kt}\text{or}\left[C\right]=\left(1-{\left[C\right]}_{0}e^{-kt}\right)$$

The resultant experimental data (concentrations of the reactant and product estimated from the NMR signal intensity) are fit to a *pseudo*-first-order process with respect to L-cys at the temperature range studied (Figure 1c,d).

The real-time monitoring of the reactant (Figure 1c) or product (Figure 1d) as a function of temperature by NMR can be used to estimate the reaction kinetics of L-CysEE. The experimental data are fit to the *pseudo*-first-order process (Equation (16))) and are also shown as continuous curves. The *pseudo*-first-order rate constants, *k*_ex_, can be estimated either from the exponential decay of the reactants (Figure 1c) or the exponential growth of the product to the maximum (Figure 1d). The rate constants can then be used in the Eyring analysis of the *pseudo*-first-order reaction kinetics, as described in Equation (11). As an example, in the case of L-CysEE, the following thermodynamic parameters are estimated (average of rate constants from both the fits): ∆*H*^‡^ = 389.6 ± 6.0 kJ/mol and ∆*S*^‡^ = *−*40.07 ± 2.0 J/(mol*·*K). The average (between 30–55 *°*C) free energy of the oxidation reaction of these molecules is ∆*G*^‡^ = 402.0 ± 20.0 kJ/mol. The estimate of thermodynamic parameters clearly shows that the free energy of the activation barrier is large enough to suggest a covalent bond formation, such as the S–S bond, due to the oxidation process.

The NMR-based approach agrees quite nicely with the rate law proposed by Wallace and Mahon, and a detailed derivation of that rate law can be found in their seminal study [23]. The kinetic events measured in this study on fresh samples of L-Cys and its ester derivatives suggest that the reaction process is mediated by the oxidation of the thiol group [25]. More importantly, the estimation of the free energy (~400 kJ/mol*·*K) from variable temperature kinetic measurements is large enough such that it establishes the formation of a covalent bond associated with the oxidation of the thiol group.

### 5.2 Intramolecular Conformational Exchange due to Partial Double Bond Rotation

The consequence of partial double bond characteristics in the amide leads to hindered rotation about the bond. The study of the restricted bond rotation in *N*,*N*-dimethylformamide (DEET) is a well-explored area in NMR spectroscopy [26–29]. *N,N*-diethyl-*m*-toluamide (*m*-DEET) is another molecule that is shown to have similar double-bond characteristics [30]. In this section, we use *m*-DEET as an example to demonstrate the measurement of molecular thermodynamic parameters. The hindered rotation, in this case, is under equilibrium between two states. The rotation can be altered by the experimental conditions, such as temperature, and thus follows an Arrhenius process.

**Figure 2:**
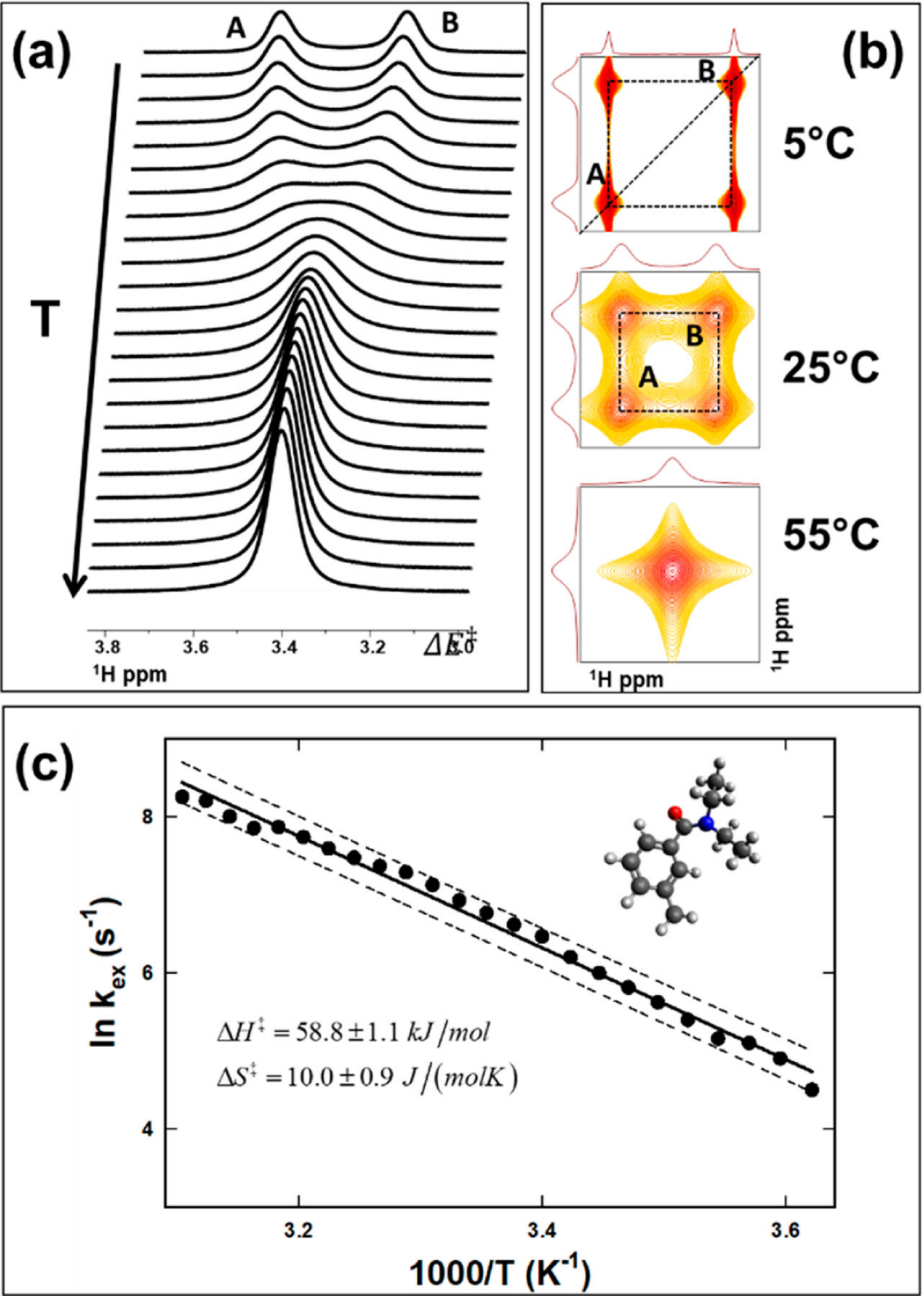
Intramolecular exchange and thermodynamics of N, N-diethyl-m-toluamide (*m*-DEET). **(a)** Spectral changes corresponding to an intramolecular exchange between two sites as a function of sample temperature, **(b)** exchange-correlation spectroscopy (EXSY) spectra of *m*-DEET at three different temperatures. In **(a)** and **(b),** the two sites ore marked as ‘A’ and ‘B.’ **(c)** Eyring plot used to determine the activation enthalpy and entropy, and the *k*_*ex*_ was estimated using the line shape analysis from the panel **(a).** The insert in panel **(c)** shows the molecular structure of *m*-DEET. Figure reproduced with permission [31].

The chemical exchange between the two states present in the same molecule, *i.e., intramolecular exchange*, introduces unique spectral features, and kinetic parameters can be extracted from the spectral features using *“line shape”* analysis. During the chemical exchange process, the two different conformations experience different magnetic environments, and hence are expected to resonate at two different frequencies, *ν*_*A*_ and *ν*_*B*_, with the difference between the two frequencies defined as ∆ = *|ν*_*A*_ *− ν*_*B*_*|*. The NMR spectral changes are directly related between ∆*ν*, and the rate of exchange *k*_*ex*_ and a schematic representation of the spectral features for a two-spin intramolecular exchange has been widely discussed in the literature (e.g., Figure 1 of [5]). The system is defined to be in *fast exchange* when $k_{ex}\gg \Delta v$, so within the NMR time scale, both exchanging sites can be differentiated spectrally, leading to > single peak . Similarly, a *slow-exchange* scenario is defined when $k_{ex}\ll \Delta v$ such that the two exchanging sites are identified at distinctly different frequencies. The *intermediate exchange* is defined when $k_{ex}\approx \Delta v$ where the distinction between the two peaks is diminished to a state of coalescence. Typically a slow-exchange condition relates to a lower sample temperature than in the ease-exchange case. In the case of *m*-DEET, Figure 2a shows the temperature dependence of the chemical exchange process with increasing temperature. At low temperatures (top trace), the partial double bond rotation is slow, leading to the slow exchange condition, while the system reaches the intermediate exchange in the middle, and subsequently reaches the fast exchange limit at high temperatures.

With the advent of two-dimensional NMR experiments, the study of chemical exchange also leapt to a different dimension [4,32,33]. The two-dimensional exchange spectroscopy (EXSY) is conducted in the same manner as the well-known nuclear Overhauser effect spectroscopy (NOESY), where the magnetization transfer between the nuclear spins is mediated via the chemical exchange process. If nuclear spins A and B undergo a conformational change between them and the NMR spectrum has distinct chemical shifts (*ν*_A_ and *ν*_B_), the chemical exchange mechanism will result in a cross peak (off-diagonal peaks) between the chemical shifts. In the EXSY spectrum, all of the exchanging sites are simultaneously explored, so that exchanges are clearly delineated. Site-to-site exchange constants can be obtained [34,35]. In spite of the value of the EXSY-based analysis, it is necessary to note that for homonuclear experiments chemical shifts between the different sites must be resolved, which may require high-field spectrometers.

Two-dimensional (2D) EXSY spectra of *m*-DEET at three different temperatures are shown in Figure 2b. At low temperature (5 *°*C), the chemical shifts of A and B are well resolved, and the cross peak (off-diagonal) indicates the exchange between the spins. In the intermediate exchange region (25 *°*C), it shows the cross peak between the exchanging spins, which are albeit broad. The two-dimensional (2D) EXSY spectra can also be used to estimate the exchange rates between the spins. For a two-site system, analytical expressions can be used to relate the exchange rate to the ratio of the intensity of the cross-peak to the diagonal peak intensities [4,36,37]. The power of the 2D EXSY experiments is realized in more complex systems such as the presence of additional exchange sites [31], subsets exchanging conformers [38] or multiple site exchanges [14,39], and the exchange between unequal populations, including intermolecular exchange (ibid., Section 5.3).

With the experimental details provided elsewhere [31], Figure 2c shows the Eyring plot for the chemical exchange mechanism for the *m*-DEET. Then, a linear fit is used to estimate the enthalpy (∆H^‡^ = 59.8 ± 1.1 kJ/mol) and entropy (∆S^‡^ = 10.9 ± 0.9 J/(mol*·*K)) of the exchange mechanism (Equation (10)). The results in Figure 2 show one of the examples of the use of NMR to determine molecular thermodynamic parameters. We have demonstrated that chemical modifications, such as *m*-DEET to *o*-DEET, can alter the conformational landscape, leading to a third sterically trapped site [31], as well as multiple sites [38]. Other mechanisms that alter molecular thermodynamic parameters include electronic, inductive, and steric effects [40,41]. The determination of the thermodynamic parameters, particularly in molecules such as DEET, has direct relevance to their utilization as potential pest repellents [42]. Therefore, this approach may provide a close correspondence between the activity of the repellent with experimental NMR measurements as well as various thermodynamic parameters.

### 5.3 Intermolecular Exchange: BMAA and its Carbonates.

The phenomenon of chemical exchange does not have to be strictly *intramolecular* in nature. When the reaction is slow, with reference to the NMR measurement time scale, distinct chemical shifts between the reactants and products (or exchanging species) may be followed by NMR spectroscopy, at times in real time. The neurotoxin BMAA (β-*N*-methylamino-L-alanine) is known to contribute to the onset of ALS/Parkinsonism-dementia complex (ALS/PDC) and the progression of other neurodegenerative diseases [43–45].

**Figure 3:**
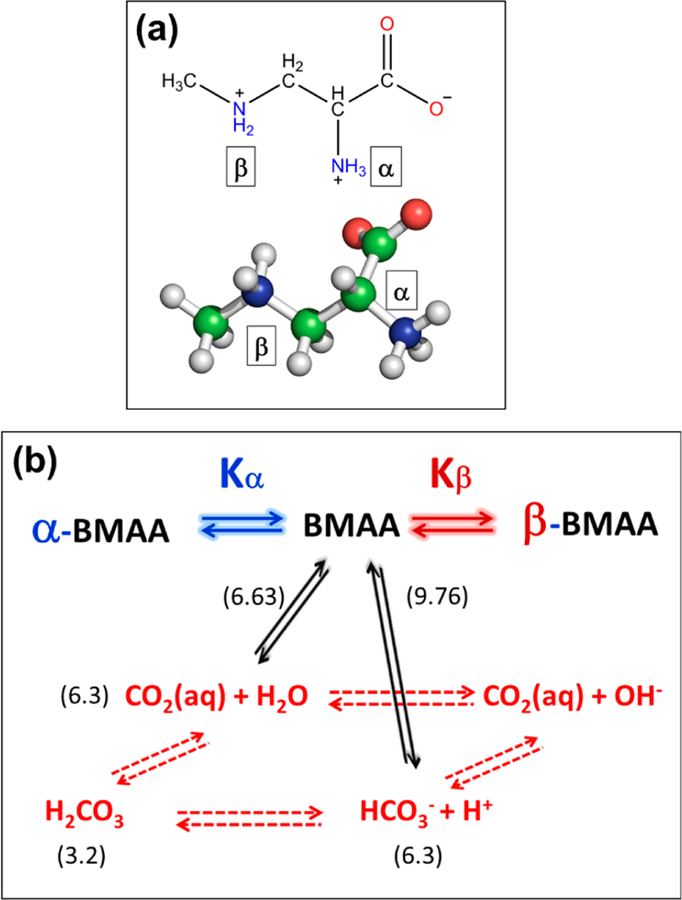
β-N-methylamino-L-alanine (BMAA) and carbamate formation. **(a)** Chemical and three-dimensional structure of BMAA. **(b)** Intermolecular exchange mechanism for the dynamic equilibria of BMAA. The equilibria between CO_2_, H_2_CO_3_, and HCO_3_^−^ are shown in red, while the equilibrium between BMAA and its carbamates are shown in black. The pKa value of each reaction is given in parenthesis. The subscripts α and β represent the primary and secondary carbamates of BMAA. Figure reproduced with permission [49].

BMAA has a secondary amine in its side chain (Figure 3a). Typically, at neutral pH, the amino group of an amino acid is protonated, and the carboxyl group is deprotonated, resulting in a neutral charge. BMAA has two amino groups in its side chain: a primary (α) and a secondary (β) amino group. This is similar to the basic amino acid lysine, but it has a different functionality. The pK_a_ values of the carboxylic group (pK_1_), the primary amine (pK_a,α_) and secondary amines (pK_a,β_), were estimated to be 2.1, 6.5, and 9.8, respectively [46]. This amine pK_a_ values were further refined [47] to be 6.63 (pK_a,α_) and 9.76 (pK_a,β_), and reproduced by the National Food Administration in Sweden [48].

However, the presence of the second amino group alters the chemistry of the carbamate formation of BMAA in a clear manner [50]. In general, the formation of carbamates follows the schematic shown in Figure 3b. Physiologically, the CO_2_ released from cellular respiration reacts with water to produce carbonic acid, which dissociates to bicarbonate and subsequently converts back to CO_2_. The presence of HCO_3_^−^ and CO_2_ can react with a deprotonated amine to produce a carbamate product (black arrows). The further reaction of HCO_3_^−^ and CO_2_ with the primary or secondary amine leads to the primary or secondary carbamylation of BMAA. The formation of primary carbamates of BMAA in the presence of bicarbonate ions was first suggested by Nunn et al. By characterizing these compounds using NMR spectroscopy [47,51,52], they first hypothesized that it was probably the formation of the second adduct of BMAA, but the second adduct could not be detected by NMR due to equipment incapability (Figure 2) . A later study by Myers and Nelson [53] used ^13^C-labeled bicarbonate and examined the formation of the BMAA/bicarbonate adducts using ^13^C experiments. The equilibrium constant of both carbamates were calculated from this study based on the relative peak intensity of the *α*-carbamate (BMAA carbamate when the primary amine binds CO_2_), β-carbamate (BMAA rarbam ate when the secondary amine binds CO_2_), and the unbound CO_2_.

Experimentally, in an aqueous solution containing BMAA and bicarbonate, it was found that 14% of BMAA was in its β-carbamate form, while 86% was in the *α*-carbamate form [53]. As discussed before, the predominance of the *α*-carbamate in comparison to the β-carbamate is due to the differences in pK_a_ between the *α*-amine and the β-amine. In effect, the differential pK_a_ values between the amines reduce the ability of the substituted secondary amine (β-amine) to be carbamylated in the presence of the primary amine (*α*-amine). The ability of the *α*-amine to be deprotonated rapidly at neutral pH allows for the nucleophilic attack of a CO_2_ and HCO_3_^−^, leading to the formation of the *α*-carbamate as opposed to the formation of tire β-carbamate from BMAA.

**Figure 4:**
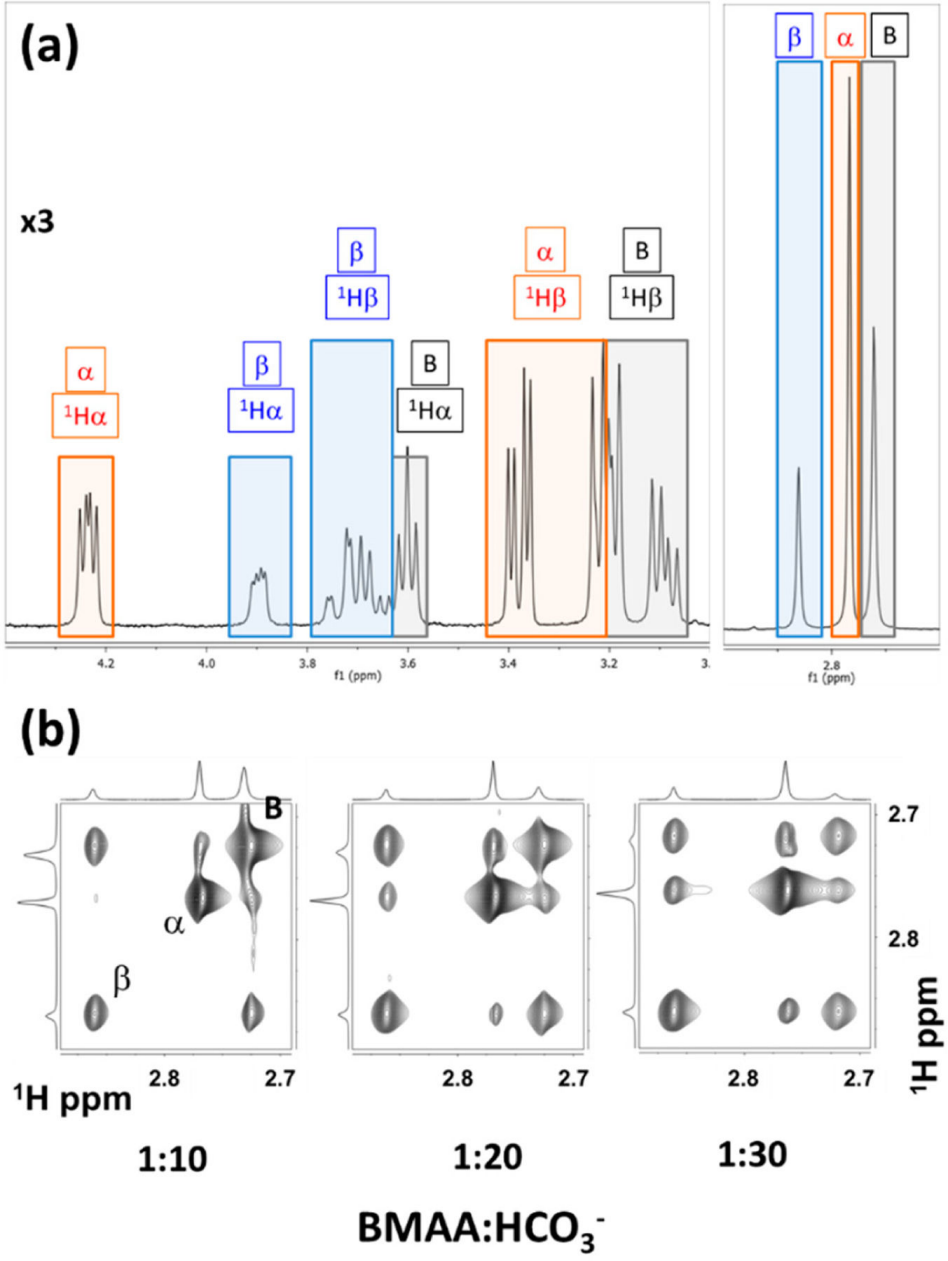
Nuclear magnetic resonance (NMR) spectral characterization of BMAA and its carbamate adducts. **(a)** Proton NMR spectrum of BMAA and its carbamates coexisting in the same sample marked by different colored boxes. **(b)** EXSY spectra off the intermolecular exchange process between BMAA and its carbamates as a function of the increasing ratio of BMAA: HCO_3_^−^. Figure reproduced under Creative Commons Attribution License from [54].

The one-dimensional ^1^H NMR spectra of BMAA, in the presence of bicarbonate ions at a ratio of 1:20, shown distinct spectral features from the free BMAA and the *α*-carbamate and β-carbamate adducts (Figure 4a) as marked by B, *α*, and β respectively. The protons 1Hα (2.72 ppm), 1Hβ (2.77 ppm), and 1Hγ (2 .86 ppm) from the BMAA, the *α*-carbamate, and the β-carbamate show the effect of conformational exchange among themselves. Two-dimensional exchange spectroscopy (EXSY) provides a compelling option to determine the equilibrium constants of nuclear spins undergoing conformation exchange [33]. The EXSY spectrum also shows the three species in the solution between the methyl protons (Figure 4b). The chemical equilibrium between BMAA and its carbamate adducts may be considered a *pseudo-equilibrium proce*ss, as the carbamate formation continues to change with an increase in the relative concentrations of the BMAA to HCO_3_^−^ ions (Figure 4b)

Following the chemical exchange process for nuclear spin relaxation between the three spins $\text{(I}=\frac{1}{2})$, the rate constants of magnetization exchange that are related to the chemical kinetics can be estimated using the well-established methods [33,55]. Using the total concentration of BMAA, which is determined upon integrating the NMR signals from the one-dimensional (1D) experiment (Figure 4a), the total concentration of carbon dioxide using the equilibration process of carbamate and carbamic acid (Figure 3), the *quasi-equilibrium* kinetics of BMAA, and its carbamates for given sample conditions (BMAA:HCO_3_^−^ concentration) can be estimated [54]. With the total concentrations of all the different species in the equilibrium process determined directly from the NMR spectrum, along with the pKa of the other reactions in the vessel [54], the EXSY data can be used to determine the thermodynamic parameters straightforwardly. The equilibrium constant for the formation of α-carbamate and β-carbamate adducts (after accounting for the protonation) is estimated as 7.6 ± 1.7 × 10^6^ and 3.1 ± 0.3 × 10^6^, respectively. The corresponding Gibbs energy (Equation (9)) (∆*G*_α_*) for the α-carbamate and β-carbamate formation are estimated to be *−*5.1 ± 1.2 kJ*·*mol^−1^, and *−*2.9 ± 0.2 kJ*·*mol^−1^, respectively. The data does not show the presence of doubly carbamylated forms of BMAA, which follows a similar observation by Davis et al. [51].

The equilibrium process of BMAA and its carbamate adducts add another dimension when these molecules interact with metal ions, particularly the divalent metals (Mn^2+^, Zn^2+^, Mg^2+^, Ca^2+^, Cu^2+^). Further investigations of this approach suggest that molecular thermodynamics parameters can be determined for various divalent metal ions using NMR spectroscopy [56].

## 6. Summary

The power of NMR-based methods to determine molecular thermodynamic parameters relies on different molecular species in a reaction process being followed with distinctive spectral signatures that can be quantitatively measured. Perhaps this feature is the unique feature of NMR spectroscopy itself. What is presented in this article is a limited experience by the author within the limited infrastructure at a predominantly teaching university. The status of NMR technology is much more powerful than the system and approaches presented here. It is left to the imagination and capacity to extend the techniques to the system of choice.
